# Analysis of vertical differentiation of vegetation in Taishan World Heritage site based on cloud model

**DOI:** 10.1038/s41598-024-61853-2

**Published:** 2024-05-13

**Authors:** Zhe Wang, Fang Han, Chuanrong Li, Weixing Shen, Zhijun Yang, Kun Li, Qi Yao

**Affiliations:** 1 School of Civil Engineering and Geomatics, Shandong University of Technology, Zibo, 255000 People’s Republic of China; 2 Mountain Tai Forest Ecosystem Research Station of State Forestry Administration/Key Laboratory of State Forestry Administration for Silviculture of the Lower Yellow River, Tai’an, 271018 People’s Republic of China; 3 Research Center for Forest Carbon Neutrality Engineering of Shandong Higher Education Institutions/Key Laboratory of Ecological Protection and Security Control of the Lower Yellow River of Shandong Higher Education Institutions, Tai’an, 271018 People’s Republic of China; 4 Mount Taishan Scenic Area Management Committee, Tai’an, 271000 People’s Republic of China; 5 Shandong Provincial Institute of Land and Space Planning, Jinan, 250013 People’s Republic of China

**Keywords:** Vertical differentiation of vegetation, Cloud model, Uncertainty, Internal structure of vertical belts, Mount Taishan, Forestry, Biogeography

## Abstract

While the forests on Mount Taishan are predominantly man-made, there is a notable vertical variation in vegetation. This study employs the method of cloud model, quantifying uncertainty (fuzziness and randomness) of things. Utilizing digital elevation model (DEM) and vegetation distribution data, we constructed elevation cloud models for Mount Taishan’s deciduous broad-leaved, temperate coniferous, and mixed coniferous-broadleaved forests. Using three numerical features of the cloud model—Expectation (EX), Entropy (EN), and Hyper-entropy (HE)—we quantitatively analyzed the macro regularity and local heterogeneity of Mount Taishan’s forests vertical distribution from the perspective of uncertainty theory. The results indicate: (1) The EX of the core zone elevation of deciduous broad-leaved forest is 716.65 m, temperate coniferous forest is 1053.51 m, and mixed coniferous-broadleaved forest is 1384.09 m. The variation range of the core zone distribution height is smaller in the mixed coniferous-broadleaved forest (EN: 53.74 m) compared to deciduous broad-leaved forest (EN: 99.63 m) and temperate coniferous forest (EN: 121.70 m). (2) The fuzziness and randomness of the distribution height of the lower extension zones of deciduous broad-leaved forest and temperate coniferous forest (EN: 75.15 m, 184.56 m; HE: 24.09 m, 63.54 m) are greater than those of the upper extension zones (EN: 44.75 m, 42.49 m; HE: 14.48 m, 13.23 m). (3) The distribution fuzziness and randomness within temperate coniferous forests exceed those of deciduous broad-leaved forests. Within the core zones, the uncertainty regarding the vertical distribution of vegetation across different aspects remains consistent, which retains the characteristic of man-made forests. However, in transition areas, there is significant disparity, reflecting the adaptive relationship between vegetation and its environment to some extent. In the upper and lower extension zones of deciduous broad-leaved forests, the EX values for the vertical distribution height of mixed coniferous and broad-leaved forests differ significantly from those of deciduous broad-leaved forests (the difference is 22.82–39.15 m), yet closely resemble those of temperate coniferous forests (the difference is 4.79–7.94 m). This suggests a trend wherein deciduous broad-leaved tree species exhibit a proclivity to encroach upon coniferous forest habitats. The elevation cloud model of vertical vegetation zones provides a novel perspective and method for the detailed analysis of Mount Taishan’s vegetation vertical differentiation.

## Introduction

Under natural conditions, any natural boundary is in reality a transition zone, which has its own two boundaries. They are, in turn, also transition zones with their own boundaries, and so on endlessly. Consequently, the localization of vegetation boundaries is in principle inexact^1^. However, we can describe the overall characteristics of vegetation distribution within transition zones by assessing the degree of boundary fuzziness. In the 1960s, scientists observed differences within vertical zones in tropical mountainous areas^2^, later termed the “internal structure theory of vertical belts”. Assuming a 1000 m wide vertical belt, the core zone spans approximately 400 m, with a 100 m upper extension, and a 500 m lower extension. The core zone represents the zone of permanent establishment for that vegetation. While the upper and lower extension zones intersect with the adjacent belts to varying degrees^3^, serving as transition areas between vertical belts. Within these transition zones, the distribution of vegetation patches from upper and lower vertical belts demonstrates a degree of randomness, while the boundaries of the vertical belts also exhibit fuzziness^4^.

The traditional research on vertical zones in mountainous areas is primarily based on field survey data, with the categorization of vertical zones relying on the distribution of vegetation types or dominant species^5,6^. Due to advancements in remote sensing and GIS technology, some scholars have attempted delineation and digital identification of vertical zones by integrating terrain data with vegetation classification data^7–10^. In recent years, some researchers have employed Geo-informatic graphic to visually represent the topographic differentiation of vegetation vertical zones^11,12^. However, there are certain shortcomings in expressing and analyzing transition zones between different vegetation types. The main reason is that most current studies on vertical zones tend to either distinctly separate upper and lower zones or consider the vertical zones boundary as a continuously changing curve along the aspect, lacking detailed analysis of transition zones and intra-zone structures at the local scale.

In order to address the inherent fuzziness and randomness in qualitative concepts, Li et al. proposed the use of cloud models^13^. Cloud models can be characterized by three numerical features—Expectation (EX), Entropy (EN), and Hyper-entropy (HE)—to represent the qualitative concept of a certain vegetation distribution height. These features reflect the spatial characteristics of vegetation vertical zone distribution: EX indicates the central tendency of its distribution height; EN comprehensively measures the range of its distribution height, indicating the uncertainty of the distribution height range; HE represents the degree of dispersion of its distribution height, with larger HE values indicating a more dispersed distribution and greater variability. Based on the cloud model approach, Mu et al. quantitatively analyzed the overall characteristics of the upper boundary (treeline) and lower boundary (timberline) distribution of the interlaced zone of the Mount Namjagbarwa^14^. They demonstrated that the cloud model could effectively reflect the uncertainty of vertical zones distribution, including the fuzziness of boundaries and the randomness of vegetation patches in transition areas.

Mount Taishan was designated as a UNESCO World Natural and Cultural Heritage Site in 1987. Prior to the 1950s, due to wars and excessive logging, the forest coverage in Mount Taishan was less than 2%. Currently, approximately 98% of Mount Taishan’s forests are artificially planted in the 1950s and 1960s^15^. Over the past 50 years, these artificial forests have undergone quasi-natural evolution, with the core zones largely retaining the attributes of the initial planted forests. The upper and lower extension zones have evolved into transition areas of different vegetation types, partially indicating the adaptive relationship between forest vegetation and the environment. Therefore, compared to the core zones, the distribution characteristics of vegetation in the transition zones may exhibit greater variation under different environmental conditions, such as topography.

In Mount Taishan, deciduous broad-leaved forests, temperate coniferous forests, and coniferous and broad-leaved mixed forests are distributed in the upper, middle, and lower regions of the mountain. The delineation between vertical belts is notably indistinct, with vegetation patches from upper or lower vertical belts scattered randomly in the transition zones. However, many studies on Mount Taishan’s vegetation vertical belts simply divide them into of broad segments of hundreds of meters, ignoring the expression and analysis of the transition zone between different vertical belts. Different scholars vary significantly in their descriptions of the number and distribution height of vertical belts on Mount Taishan. Ma, et al. divided vegetation vertical belts of Mount Taishan into warm temperate deciduous forest zone (200–600 m), temperate deciduous broad-leaved forest subzone (600–800 m), cold temperate deciduous broad-leaved forest subzone (800–1000 m), and coniferous and broad-leaved mixed forest zone (1000–1500 m) from bottom to top^16^. Liang, et al. think that the deciduous broad-leaved forest is mainly distributed at an altitude of 550–1000 m, the temperate evergreen coniferous forest is mainly distributed at an altitude of more than 1000 m, and the distribution of coniferous and broad-leaved mixed forests is not concentrated^17^. While the lack of uniform criteria contributes to the inconsistency in results^18^, a deeper issue also lies in the inadequacy of the methods currently employed in mountain vegetation vertical zones research, failing to exhibit its complexity. Therefore, using the cloud model method to analyze the vegetation vertical belts of Mount Taishan can quantitatively describe the uncertainty of each vertical belt’s distribution and its transition zones. This approach enhances the expression of the complexity of its distribution, deepening people’s understanding of the distribution pattern of Mount Taishan’s vegetation vertical belts.

In this paper, the research on vegetation vertical belts in Mount Taishan is primarily divided into two parts: (1) The main distribution areas of the upper extension zone, core zone, and lower extension zone of deciduous broad-leaved forest, temperate coniferous forest, and coniferous and broad-leaved mixed forest are delineated. Subsequently, elevation cloud models for each vertical belt are established to quantitatively analyze the uncertainty of the distribution of vegetation within core zones and transition zones. (2) Based on the altitude and aspect of the study area, analysis units were delineated, and elevation cloud models were established for each unit. From the local scale, the uncertainty of the distribution of vegetation vertical belts under different terrain conditions was quantitatively analyzed, and the adaptation relationship between forest vegetation and environment in Mount Taishan was explored. This approach provides a new perspective and method for analyzing the complexity of Mount Taishan’s vegetation vertical zones distribution in detail.

## Materials and methods

### Study area

Mount Taishan is situated on the eastern side of the North China Plain, in the central part of Shandong Province (36° 10′ ~ 36° 20′ N, 117° 0′ ~ 117° 10′ E). The terrain slopes from south to north, with its main peak, Yuhuangding Peak, reaching an elevation of 1532.5 m. The relative height difference between the foothill plain and the summit is 1391 m. Mount Taishan is located in the temperate monsoon climate zone, resulting in distinct climatic differences across various terrains and altitudes. The annual average temperature at the mountain’s summit is 5.3 °C, which is 7.5 °C lower than that at the foothill. The summit receives an annual average precipitation of 1124.6 mm, 1.5 times higher than the precipitation at the mountain’s base. At the foot of Mount Taishan, the annual average temperature is 12.8 °C, with an annual average precipitation of 715.0 mm, which increases with the elevation gain. The winter season on Mount Taishan is relatively long, lasting 150 days with a freezing period, and the lowest recorded temperature at the summit reaches − 27.5 °C^19^.

**Figure 1:**
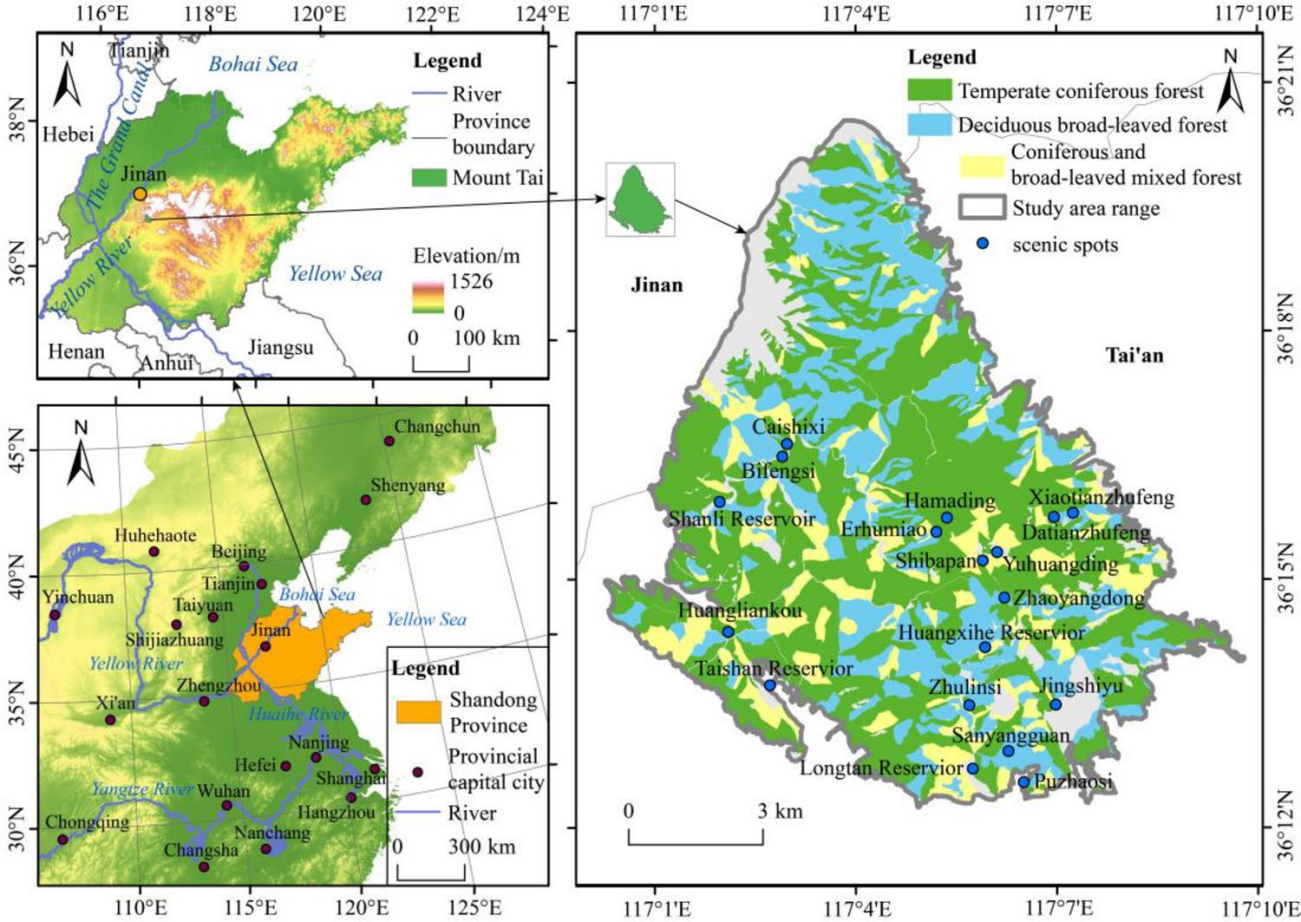
The study area location and forest vegetation distribution.

Mount Taishan’s forest types primarily include coniferous forests, broad-leaved forests, and mixed coniferous-broadleaved forests. Among them, the coniferous forests are predominantly composed of temperate coniferous species, including Chinese pine (*Pinus tabulaeformis*), oriental arborvitae (*Platycladus orientalis*), Red pine (*Pinus densiflora Sieb. et Zucc.*), Black pine (*Pinus thunbergii Parl.*), and Armand pine (*Pinus armandii Franch.*). Mount Taishan lacks typical cold-temperate coniferous vegetation, but it includes introduced cold-temperate tree species from other regions, such as Japanese larch (*Larix kaempferi*), North China larch (*Larix gmelinii var. principis-rupprechtii*), and Dahurian larch (*Larix gmelinii (Rupr.) Kuzen*). Broad-leaved forests are dominated by species such as sawtooth oak (*Quercus acutissima Carruth.*), Chinese cork oak (*Quercus variabilis Bl.*), black locust (*Robinia pseudoacacia*), and maple-leaved viburnum (*Acer mono Maxim*)^20^. Figure 1 provides an overview of the study area, illustrating the forest types and their distribution.

### Data sources

#### DEM data

The digital elevation model (DEM) data was provided by the Forest Resource Management Station of Mount Taishan Scenic Area Management Committee, with a spatial resolution of 2.5 m.

#### Forest resource inventory “One Map” data

Forest Resource Inventory “One Map” data was provided by the Forest Resources Management Station of Mount Taishan Scenic Area Management Committee. The survey conducted in 2014 includes attribute information about forests, such as dominant tree species and tree species composition.

### Cloud model

Following the concept of cloud model proposed by Li et al.^13^, We construct elevation cloud models for the vertical vegetation zone of Mount Taishan. Taking the upper extension zone of deciduous broad-leaved forest as an example, where U represents a quantitative domain of the upper extension zone elevation of deciduous broad-leaved forest expressed by accurate numerical values, with X ⊆ U, and T donates the qualitative concept of the upper extension zone elevation in space. If x(x ∈ X) is a numerical example of the upper extension zone elevation, C_T_(x) maps the example x to a metric representing its relative belonging to the upper extension zone (represented by T) on a scale of 0 to 1, that is C_T_(x) ∈ [0, 1], where 1 indicates that the example belongs and 0 indicates that it does not. And C_T_(x) is a random number with a stable tendency (Eq. 1). Subsequently, the mapping of T from domain U to interval [0, 1] in data space is termed as a “cloud”. Each discrete elevation point within the upper extension zone of deciduous broad-leaved forest is a cloud droplet within the cloud model.

1 $$ C_{T} \left( x \right):U \to \left[ {0,1} \right] \forall x \in X\left( {X \subseteq U} \right),x \to C_{T} \left( x \right). $$

The three numerical features of the cloud model—Expectation (EX), Entropy (EN), and Hyper-Entropy (HE)—can reflect the overall characteristics of the upper extension zone elevation of deciduous broad-leaved forest. EX is the value that can best represent the overall situation of its elevation, indicating the central tendency of distribution height. EN provides a holistic measure of the variation range in elevation, thereby indicating the fuzziness inherent in the distribution. In general, higher EN values correspond to increased fuzziness within the distribution. Meanwhile, HE quantifies the uncertainty within EN, reflecting the degree of variation in the elevation of the upper extension zone within the deciduous broad-leaved forest. That is, the larger the value of HE, the greater the degree of dispersion of the elevation, the greater the randomness.

### Construction of elevation cloud models of the upper extension zone, core zone and lower extension zone of vertical belts in Mount Taishan

#### Forest type classification and area proportion statistics

**Figure 2:**
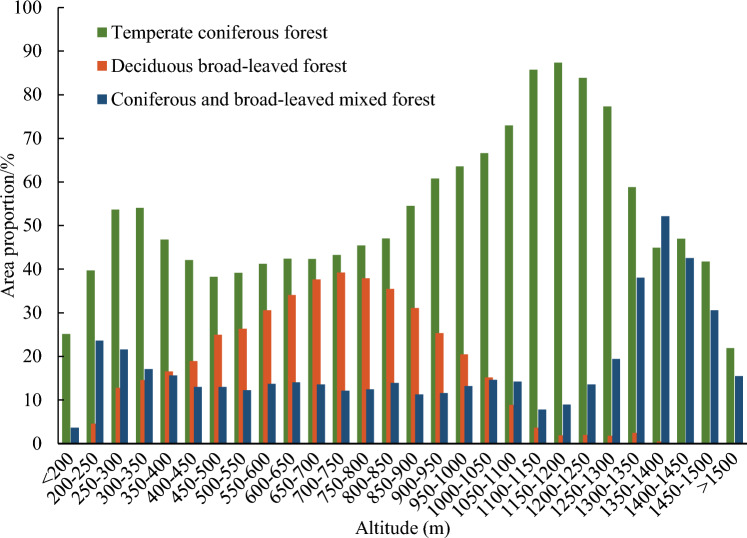
The area proportions of deciduous broad-leaved, temperate coniferous, mixed coniferous-broadleaved forests at different altitudes.

Based on the “dominant tree species” and “tree species composition” fields of attribute table of the Forest Resource Inventory “One Map” and following the guidelines outlined in the “Construction of Forest Resource Dynamic Monitoring Information System in Shandong Province—Operational Rules for Forest Resource Survey”, plots where the proportion of broad-leaved tree species or coniferous tree species exceeded 7 (up to 10) were categorized as deciduous broad-leaved forest or temperate coniferous forest, respectively. Plots with a different composition were classified as mixed coniferous-broadleaved forest. Subsequently, using ArcGIS software, we reclassified the DEM, into 28 categories by setting elevation intervals at 50 m. Then we calculated the proportions of deciduous broad-leaved, temperate coniferous, and mixed coniferous-broadleaved forests within each elevation interval. These proportions are illustrated in Fig. 2.

#### Division of core zone, upper extension zone and lower extension zone, and sample points extraction

Deciduous broad-leaved, temperate coniferous and mixed coniferous-broadleaved forests exhibit distribution across the upper, middle, and lower parts of Mount Taishan, with notably unclear boundaries of the vertical zones. Most geobotanists accepted the concept of vegetation cover changes gradually, forming communities based less on distinct boundaries and more on gradients of quantitative change^21^. Therefore, in this study, we determined the main distribution areas of their respective core zone, upper extension zone, and lower extension zone based on the changing gradients in the area proportions of deciduous broad-leaved, temperate coniferous and mixed coniferous-broadleaved forests. As shown in Fig. 2, the proportions of deciduous broad-leaved and temperate coniferous forests initially increase and then decrease with the elevation. Deciduous broad-leaved forest primarily occurs at altitudes of 250–1050 m, covering 12.81 to 39.21% of the area. The proportion in other areas is small (0.47 to 8.85%). Temperate coniferous forest is mainly distributed at altitudes of 200–1500 m, comprising 39.71 to 87.36% of the area. The proportion in other areas is 21.88 to 25.12%, showing significant differences. Notably, a small concentrated area of mixed coniferous-broadleaved forest exists at 1300–1500 m, ranging from 30.6 to 52.13%, significantly different from other areas (3.65 to 23.66%).

Therefore, this study established the average of area proportion of deciduous broad-leaved forest in the range of 250–1050 m (16 altitude intervals) as the thresholding (26.32%). Regions with a proportion greater than 26.32% were identified as the main distribution areas of the core zone, while those with a proportion lower than 26.32% were classified as the primary distribution areas of the upper and lower extension zones. Similarly, the average of area proportion of temperate coniferous forest in the range of 200–1500 m (26 altitude intervals) was set as the thresholding (54.64%), following the same division method as for deciduous broad-leaved forest. Given the limited area of concentrated distribution for mixed coniferous-broadleaved forest, a threshold of 30% was adopted, using the same division method as for deciduous broad-leaved forest. However, the projected area of mixed coniferous-broadleaved forest above 1500 m is too small, with fewer than 3 sample points, insufficient to support the construction of cloud models, and its impact on the research results can be neglected. Therefore, no analysis was performed for this area. The division results are shown in Table 1.

**Table 1:** Internal structure and sample points of deciduous broad-leaved, temperate coniferous and mixed coniferous-broadleaved forests.

Forests type	Thresholding	Internal structure	Area proportion/%	The altitude range of the sample points (m)	Number of sample points
Deciduous broad-leaved forest	26.32%	Upper extension zone	< 26.32	> 900	74
		Core zone	26.32–39.21	550–900	568
		Lower extension zone	< 26.32	< 550	198
Temperate coniferous forest	54.64%	Upper extension zone	< 54.64	> 1350	28
		Core zone	54.64–87.36	900–1350	334
		Lower extension zone	< 54.64	< 900	1120
Mixed coniferous-broadleaved forest	30%	Core zone	30–52.13	1300–1500	23
		Lower extension zone	< 30	< 1300	436

Finally, we employed ArcGIS software to establish a 200 m point grid across the study area. Subsequently, we extracted sample points data of core zone, upper extension zone and lower extension zone of deciduous broad-leaved, temperate coniferous and mixed coniferous-broadleaved forests. This was achieved by using the “Extract Values to Points” tool to match the elevation values.

#### Construction of elevation cloud models

We construct a cloud model for each zone in Table 1. Based on a software named MATLAB2019, the three digital characteristics Ex, En and He of the cloud model are obtained by using the reverse cloud generator, and the cloud droplets are obtained to generate the cloud maps by using the forward cloud generator.

Reverse cloud generator algorithm steps are as follows:

*Step 1*: Input sampling point elevation data.

*Step 2*: Calculate the mean $\overline{X }$ and variance $S$ of the sample data.

2 $$\overline{X }=\frac{1}{n}\sum_{i=1}^{n}{x}_{i},$$

3 $$S=\frac{1}{n-1}\sum_{i=1}^{n}{\left({x}_{i}-\overline{X }\right)}^{2},$$

where ${x}_{i}$(*i* = 1,2,$\cdots$,*n*) is the sample value and *n* is the number of sample points.

Step 3: Calculate Ex.

4 $$Ex=\overline{X },$$

Step 4: Calculate En.

5 $$En=\sqrt{\frac{\pi }{2}}\times \frac{1}{n}\sum_{i=1}^{n}\left|{x}_{i}-Ex\right|,$$

Step 5: Calculate He.

6 $$He=\sqrt{S-{En}^{2},}$$

Forward cloud generator algorithm steps are as follows:

*Step 1*: Set the number of generated cloud droplets *m*.

*Step 2*: Generate a normal random number ${En}{\prime}$ with *En* as the expected value and *He* as the standard deviation.

*Step 3*: Generate a normal random number *x* with *Ex* as the expected value and ${En}{\prime}$ as the standard deviation.

*Step 4*: Calculate the membership degree ${C}_{T}\left(x\right)$ corresponding to *x*.

7 $${C}_{T}\left(x\right)={e}^{-\frac{{\left(x-Ex\right)}^{2}}{2{\left({En}{\prime}\right)}^{2}},}$$

*Step 5*: The *x* with ${C}_{T}\left(x\right)$ is a cloud droplet of the cloud model.

*Step 6*: Repeat Step 2 ~ Step 5 until the number of cloud droplets generated is *m*.

*Step 7*: The cloud model is drawn with *x* as abscissa and ${C}_{T}\left(x\right)$ as ordinate.

### Construction of elevation cloud model of different terrain conditions of vertical belts in Mount Taishan

We classified the elevation and aspect of the study area. Elevation classification is based on Table 1, which is divided into < 550 m, 550–900 m, 900–1100 m, 1100–1350 m,  > 1350 m. The aspect is divided into sunny slope (112.5°–292.5°) and shady slope (0°–112.5°, 292.5°–360°, flat slope). As shown in Table 2, the study area is divided into 10 analysis units. In addition, the reason for choosing aspect instead of slope is that forests of Mount Taishan has a significant distribution in different aspect: the proportion of temperate coniferous forest species on shady slopes is relatively large, and the proportion of deciduous broad-leaved tree species on sunny slopes is relatively large. Finally, the same as the construction steps of internal structure cloud model of vertical belts, the sampling points are extracted and the elevation values are matched. The elevation cloud model of deciduous broad-leaved forest, temperate coniferous forest and mixed coniferous-broadleaved forest is established in each analysis unit. In addition, due to deciduous broad-leaved forests are basically not distributed above an altitude of 1100 m, there were no sampling points in this area.

**Table 2:** Sampling points in different analysis units of vertical belts in Mount Taishan.

Altitude (m)	Forests type	Number of sample points	
		Sunny slope	Shady slope
< 550	Deciduous broad-leaved forest	137	64
	Temperate coniferous forest	227	190
	Mixed coniferous-broadleaved forest	87	62
550–900	Deciduous broad-leaved forest	339	231
	Temperate coniferous forest	270	440
	Mixed coniferous-broadleaved forest	116	115
900–1100	Deciduous broad-leaved forest	51	31
	Temperate coniferous forest	106	131
	Mixed coniferous-broadleaved forest	28	29
1100–1350	Temperate coniferous forest	57	54
	Mixed coniferous-broadleaved forest	9	12
> 1350	Temperate coniferous forest	11	17
	Mixed coniferous-broadleaved forest	8	11

## Results

### Vertical distribution analysis of dominant tree species

**Figure 3:**
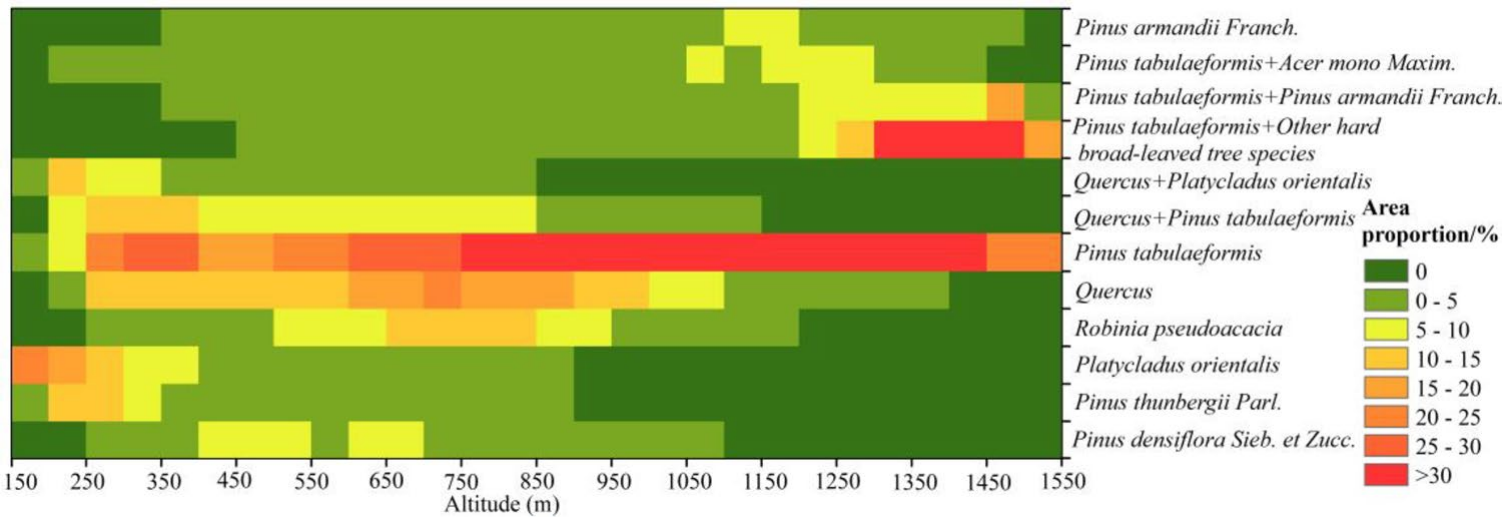
The area proportion of dominant tree species at different altitudes.

As shown in Fig. 3 and Table 3, the dominant broad-leaved tree species on Mount Taishan are *Quercus* and *Robinia pseudoacacia*. *Quercus* predominates between altitudes of 250 to 1100 m, covering 6.26 to 20.94% of the area. Meanwhile, *Robinia pseudoacacia* mainly inhabits altitudes ranging from 500 to 950 m, representing 6.31 to 12.71% of the area. Coniferous tree species such as *Pinus tabulaeformis*, *Pinus densiflora Sieb. et Zucc.*, *Pinus thunbergii Parl.*, *Platycladus orientalis*, and *Pinus armandii Franch.*, et al. exhibit diverse distribution patterns. *Pinus tabulaeformis*, for instance, dominates areas above 250 m, reaching up to 73.91%, with a notable concentrated distribution at altitudes between 750 and 1450 m, where the area percentage exceeds 30%. *Pinus densiflora Sieb. et Zucc.*, *Pinus thunbergii Parl.*, and *Platycladus orientalis* are more prevalent below 700 m, while *Pinus armandii Franch.* is primarily found above 1100 m. The mixed coniferous-broadleaved forest mainly consists of *Pinus tabulaeformis* and broad-leaved species. Below 850 m above sea level, the proportion of *Quercus* + *Pinus tabulaeformis* and *Quercus* + *Platycladus orientalis* mixed forests occupy a larger proportion (5.95 to 13.46%). Above 1050 m, predominant mixed forests include *Pinus tabulaeformis* + *Pinus armandii Franch.*, *Pinus tabulaeformis* + *Acer mono Maxim.*, and *Pinus tabulaeformis* + Other hard broad-leaved tree species.

**Table 3:** Classification of dominant tree species.

Forests type	Dominant tree species
Deciduous broad-leaved forest	*Robinia pseudoacacia*
	*Quercus*
Temperate coniferous forest	*Pinus densiflora Sieb. et Zucc*
	*Pinus thunbergii Parl*
	*Platycladus orientalis*
	*Pinus tabulaeformis*
	*Pinus armandii Franch*
	*Pinus tabulaeformis* + *Pinus armandii Franch*
Mixed coniferous-broadleaved forest	*Pinus tabulaeformis* + *Acer mono Maxim*
	*Pinus tabulaeformis* + Other hard broad-leaved tree species
	*Quercus* + *Platycladus orientalis*
	*Quercus* + *Pinus tabulaeformis*

### Uncertainty analysis of the distribution height of the internal structure of vegetation vertical belts in Mount Taishan

#### Deciduous broad-leaved forest

**Figure 4:**
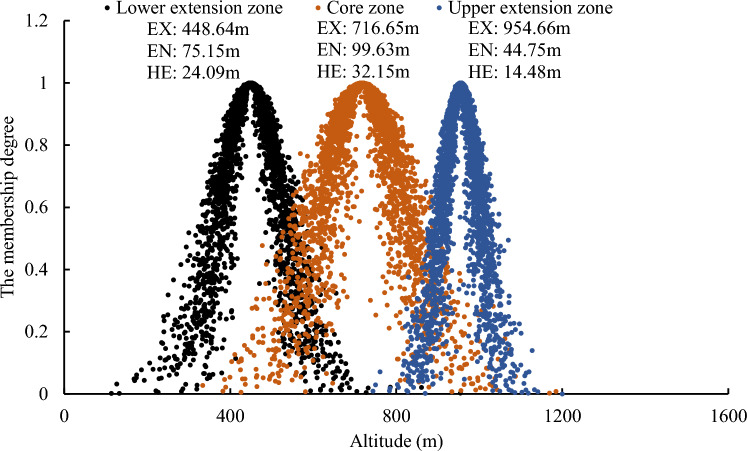
Elevation cloud model of broad-leaved forest.

As shown in Fig. 4, for the core zone of the deciduous broad-leaved forest, the Expectation (EX) measures 716.65 m, indicating that the center of its distribution height. The Entropy (EN) and Hyper-Entropy (HE) stand at 99.63 m and 32.15 m, respectively. Notably, the dominant tree species are *Quercus* and *Robinia pseudoacacia*. The center of the distribution height of the upper extension zone is 954.66 m, accompanied by EN and HE values of 44.75 m and 14.48 m, respectively, with *Quercus* as the dominant tree species. Contrarily, the lower extension zone’s distribution height centers at 448.64 m. Both its EN (75.15 m) and HE (24.09 m) exceed those of the upper extension zone. This difference suggests that in comparison to the upper extension zone, the lower extension zone has a broader range and greater variability in distribution height, demonstrating higher degrees of fuzziness and randomness in its distribution.

#### Temperate coniferous forest

**Figure 5:**
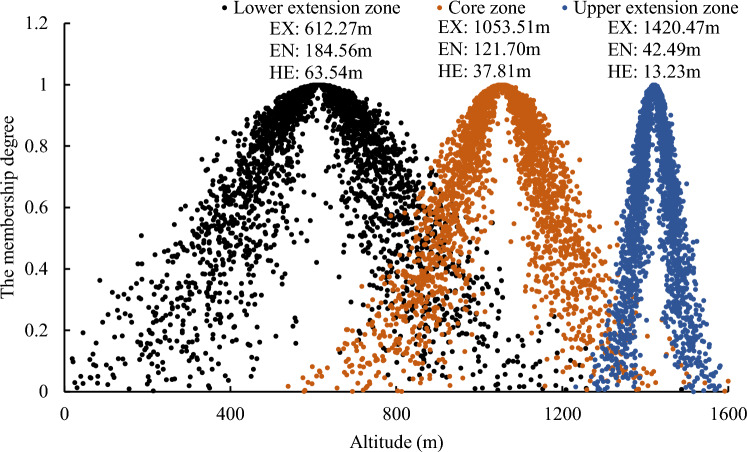
Elevation cloud model of coniferous forest.

In the core zone of the temperate coniferous forest, the center of the distribution height measures 1053.51 m, accompanied by EN and HE values of 121.70 m and 37.81 m, respectively, with *Pinus tabulaeformis* as the dominant tree species. Moving to the upper extension zone, the distribution height’s center is at 1420.47 m, with EN and HE values of 42.49 m and 13.23 m, respectively, with dominance from *Pinus tabulaeformis* and *Pinus armandii Franch*. As depicted in Fig. 5, it can be observed that, compared to the upper extension zone, the lower extension zone’s cloud model exhibits a broader range and greater variability in the distribution of cloud droplets (EN: 184.56 m, HE: 63.54 m). This difference indicates that the lower extension zone within the temperate coniferous forest has a wider variation range of distribution height, with higher fuzziness and randomness in its distribution.

#### Mixed coniferous-broadleaved forest

**Figure 6:**
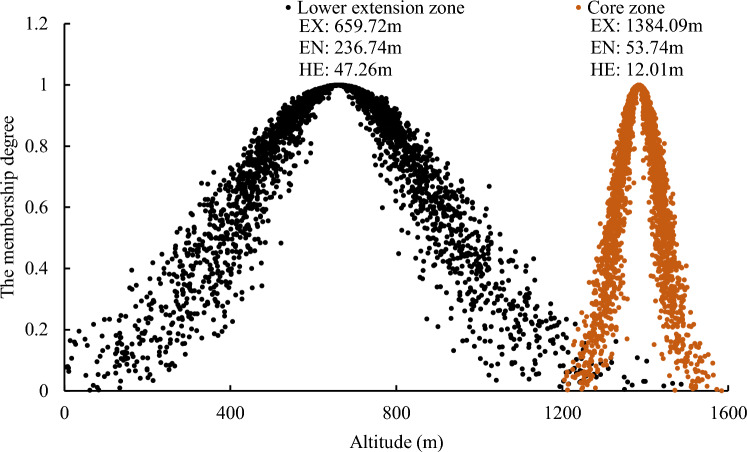
Elevation cloud model of mixed coniferous-broadleaved forest.

For the core zone of the mixed coniferous-broadleaved forest, the EN value (53.74 m) notably contrasts with that of the deciduous broad-leaved forest (99.63 m) and the temperate coniferous forest (121.70 m). This indicates a narrower variation range in distribution height within its core zone, exhibiting less pronounced exhibiting. However, the core zone displays a relatively higher distribution height (EX: 1384.09 m), primarily characterized by mixed forests of *Pinus tabulaeformis* and hard broad-leaved tree species (excluding *Quercus* and *Robinia pseudoacacia*), such as Siberian alder (*Alnus sibirica*), and Amur cork tree (*Phellodendron amurense Rupr*). From Fig. 6, it can be observed that the lower extension zone of the coniferous and broadleaved mixed forest has a large range of distribution height (EN: 236.74 m), and the distribution height shows significant variability (HE: 47.26 m), mainly consisting of mixed forests of *Pinus tabulaeformis* and *Quercus*.

### Uncertainty analysis of vegetation vertical belts in Mount Taishan in different terrains

As indicated in Table 4, across various topographies, the values of EN and HE in the elevation cloud models of temperate coniferous forests are higher, while those of deciduous broad-leaved forests are lower. This suggests that temperate coniferous forests display greater fuzziness and randomness in distribution compared to deciduous broad-leaved forests.

**Table 4:** The digital characteristics of elevation cloud model of vegetation vertical belts in different analysis units of Mount Taishan.

Altitude (m)	Forests type	Sunny slope			Shady slope		
		EX (m)	EN (m)	HE (m)	EX (m)	EN (m)	HE (m)
< 550	Deciduous broad-leaved forest	407.51	67.92	17.56	438.58	53.15	8.19
	Temperate coniferous forest	378.51	79.12	24.22	404.91	64.89	16.46
	Mixed coniferous-broadleaved forest	370.57	83.01	26.26	399.43	70.13	10.46
550–900	Deciduous broad-leaved forest	700.89	110.01	34.51	700.58	108.92	34.73
	Temperate coniferous forest	695.54	124.66	48.07	706.86	116.89	40.92
	Mixed coniferous-broadleaved forest	701.82	122.30	46.43	686.65	109.73	33.66
900–1100	Deciduous broad-leaved forest	963.56	46.08	12.13	965.84	57.76	17.90
	Temperate coniferous forest	987.51	60.86	21.50	983.87	57.78	19.57
	Mixed coniferous-broadleaved forest	992.33	56.72	19.61	988.66	57.10	20.46
1100–1350	Temperate coniferous forest	1182.76	57.93	18.60	1188.50	59.57	21.50
	Mixed coniferous-broadleaved forest	1213.16	64.36	21.73	1218.48	61.54	17.06
> 1350	Temperate coniferous forest	1377.64	64.09	26.90	1382.60	59.60	21.02
	Mixed coniferous-broadleaved forest	1373.57	48.58	16.77	1389.07	56.38	16.27

**Figure 7:**
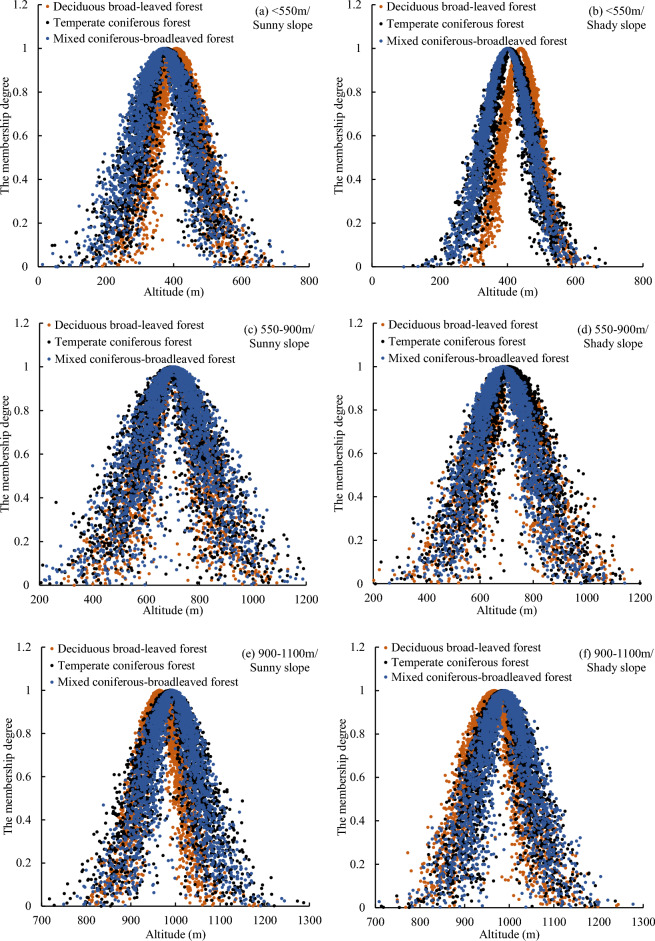

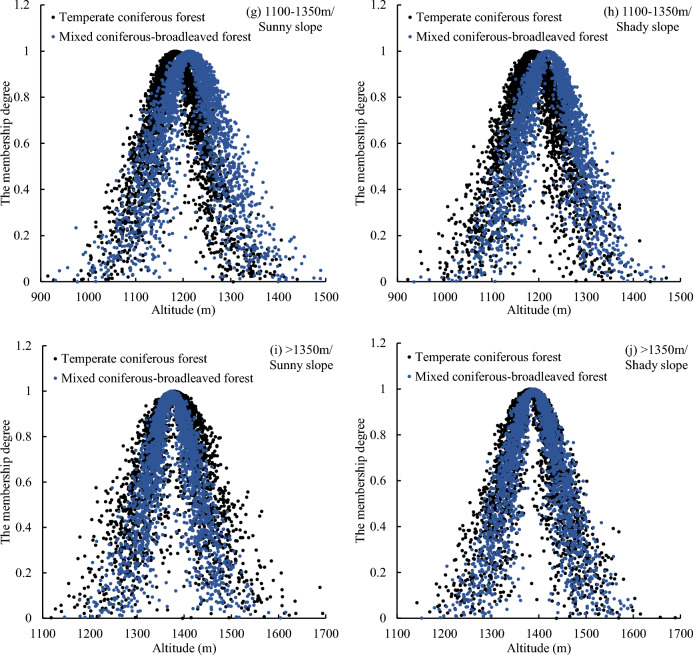
Elevation cloud models of vegetation vertical belts in different analysis units of Mount Taishan.

As shown in Fig. 7 and Table 4, the distribution characteristics of deciduous broad-leaved forests at different aspects are relatively consistent at an altitude of 550–900 m, with EX values of the elevation cloud model are 700.58–700.89 m, EN are 108.92–110.01 m, and HE are 34.51–34.73 m. However, below 550 m and between 900 and 1100 m, the distribution characteristics of deciduous broad-leaved forests vary significantly across different aspects. At altitudes of 900–1100 m and 1100–1350 m, the distribution characteristics of temperate coniferous forests across different aspects are relatively consistent, with EX values of the elevation cloud model are 983.87–987.51 m and 1182.76–1188.50 m, EN are 57.78–60.86 m and 57.93–59.57 m, and HE are 19.57–21.50 m and 18.60–21.50 m. However, below 900 m and above 1350 m, the distribution characteristics of temperate coniferous forests vary significantly across different aspects. It can be observed that in the core zone, the distribution characteristics of deciduous broad-leaved forests and temperate coniferous forests on different aspects are similar, while in the upper and lower extension zones, the differences are significant. Based on this discovery, we can speculate that the core zones of the vertical belt still retain the characteristics of artificial forests. Because the guiding ideology of afforestation in Mount Taishan is to establish forests first and then optimize the forest structure, the distribution characteristics of artificial forests may be similar under different environmental conditions. But in the upper and lower extension zones, after decades of near-natural succession, the adaptability of vegetation to the environment is reflected. For example, to capture more heat, the uncertainty of vegetation distribution on the shady slopes is smaller, with vegetation concentrated in higher terrain areas, while the uncertainty of vegetation distribution on the sunny slopes is greater. In adddition, the reason why coniferous and broad-leaved mixed forest does not have this law is mainly because it has only a small range of concentrated distribution.

As shown in Fig. 7a–f, at the altitude of 550–900 m, the EX of the distribution height of deciduous broad-leaved forest, temperate coniferous forest and coniferous and broad-leaved mixed forest is very close. At the altitude of < 550 m and 900–1100 m, the EX of the distribution height of deciduous broad-leaved forest and coniferous and broad-leaved mixed forest is quite different, and the difference is 22.82–39.15 m, while the EX of the distribution height of temperate coniferous forest and coniferous and broad-leaved mixed forest is close, and the difference is 4.79–7.94 m. This may be related to the invasion of deciduous broad-leaved tree species in Mount Taishan to coniferous forests. Meng^15^ found that broad-leaved tree species such as *Robinia pseudoacacia* have a tendency to expand to pine forests. From 2000 to 2016, the proportion of pine trees in Mount Taishan forest decreased from 55.69 to 50.22%, the proportion of *Robinia pseudoacacia* increased from 10.15 to 13.75%, the area increased by 421.85 ha, and the proportion of *Quercus* also increased by 1.77%^22^. In addition, the difference of EX between deciduous broad-leaved forest and coniferous and broad-leaved mixed forest is 36.94–39.15 m below 550 m, and 22.82–28.77 m at 900–1100 m, which indicates that deciduous broad-leaved tree species have a greater degree of invasion to coniferous forest in low altitude areas.

## Discussion

### Division method discussion for core zone, upper extension zone and lower extension zone

Most forests in Mount Taishan are artificially cultivated, the vertical distribution of vegetation zones is significantly influenced by human activities, but it inevitably hides the patterns of natural succession. After several decades of near-natural succession, the core zones within the vertical zones have largely retained the attributes of artificial forests. Conversely, the upper and lower extension zones, serving as transition areas between different vegetation types, somewhat reflect the adaptive relationship between vegetation and the environment. However, the study area’s relatively limited size poses challenges in obtaining a sufficient quantity of meteorological data points essential for comprehensive research. Additionally, comprehending how local-scale vegetation adapts to the terrain requires long-term field observations. Therefore, this study has yet to analyze the relationship between the upper and lower extension zones of various forest types and environmental factors concerning species-environment adaptation.

Theory regarding the internal structure within vertical zones suggests that the core zone occupies around 40% of the vertical width, the upper extension zone is approximately 10%, and the lower extension zone is about 50%^3^. This study divided the main distribution areas of the core zone, upper extension zone and lower extension zone based on the average area proportions of each forest type. Consequently, cloud models were established for the temperate coniferous forest, the proportion of the distribution height variation range approximates 35% for the core zone, 12% for the upper extension zone, and 53% for the lower extension zone—close to the theoretical values. However, for the deciduous broadleaf forest, the proportions are 45%, 20%, and 35%, respectively, slightly deviating from the theoretical values. Mount Taishan is located in the warm temperate humid semi-humid monsoon climate zone, categorized within the warm temperate deciduous broadleaf forest zone. The primary tree species in the base belt should typically be positive winter deciduous broad-leaved tree species (Below 500 m above sea level)^23^. Nevertheless, due to human influence, broadleaf tree species are mainly distributed in the middle part of Mount Taishan, between 500 and 1000 m in altitude, with fewer distributions at lower altitudes and practically absent below 250 m. Thus, if in a natural succession state, the proportion of the lower extension zone of deciduous broadleaf forest in Mount Taishan should also approach 50%. The research results can reflect the internal variations within Mount Taishan’s vegetation vertical zones. Hence, this study contends that using the mean value of area proportions as a threshold to delineate the core zone, upper extension zone, and lower extension zone within Mount Taishan’s vertical belts is both practical and aligns with observed patterns.

### Discussion on the near-natural succession of the upper and lower extension zones in the vertical vegetation belts of Mount Taishan

The research findings of this study indicate significant differences in the distribution characteristics of vegetation on different aspects within the transition zone, which we attribute to natural succession. However, due to a lack of supporting data (such as the initial planting pattern of trees), it cannot be ruled out that the planting pattern of trees during afforestation is the case. Nevertheless, through field surveys and analysis of tree appearance characteristics, it can be preliminarily concluded that the difference in vegetation distribution on different aspects within the transition zones are the result of understory regeneration and natural selection. For example, on shady slopes, vegetation can obtain more heat in higher terrain areas, so understory regeneration is faster.

In the extension zones of deciduous broad-leaved forest, there is a trend of broad-leaved tree species expanding to coniferous forest. Research has shown that *Robinia pseudoacacia* can spread through root shoots into dense and evenly planted *Pinus tabulaeformis* forest, forming the dominant companion understory. However, if there are forests gaps or skylights resulting from dead trees, *Robinia pseudoacacia* can occupy the space and quickly grow into the upper canopy, forming a mixed forest with the original dominant trees^24^. Moreover, in recent years, Mount Taishan has experienced overall increase in average annual temperature and a decrease in annual precipitation, resulting in dry and windy springs, hot summers, and cold, less snowy winters^25^. In response to the drought in spring and the continuous high temperature in summer, the adaptability of pines is not as good as that of *Robinia pseudoacacia*^15^^,^^26,27^. The rising temperatures have expanded the range and quantity of pests and diseases in the coniferous forests, worsening their impact^28^. Pest Invasions weaken the growth of pine trees, even causing death, creating favorable conditions for the expansion of broadleaf tree species. As altitude increases, temperatures gradually decrease, and the relatively infertile soil becomes more suitable for the growth of coniferous tree species. Above 1050 m in altitude, broadleaf tree species are only scattered. Consequently, deciduous broad-leaved forest has a greater expansion trend in low altitude areas, and its distribution range of lower extension zone (EN: 75.15 m) is greater than that of upper extension zone (EN: 44.75 m).

Temperate coniferous forests are the most widely distributed in Mount Taishan, mostly consisting of artificially cultivated coniferous pure forests, with pines being predominant. However, below 1000 m in altitude, pines are less adaptable to the environment than broadleaf tree species, and their proportion gradually decreases over time. This leads to the formation of a larger lower extension zone for temperate coniferous forests (EN: 184.56 m). Above 1500 m in altitude, only 22% of temperate coniferous forests and 15% of coniferous-broadleaf mixed forests are distributed. The significant decrease in forest coverage in the area, besides the influence of the mountain topography, may also be attributed to the adverse effects of prolonged low temperatures and strong light during the winter. Researchers believe that the height of the timberline is determined by the temperature during the growing season, not the winter low temperatures^29^. Körner proposed that the height of the temperate timberline is consistent with the average temperature of the warmest month being 10°C^1^, while the average temperature of the warmest month on the southern slope of Mount Taishan is 18.42–26.81 °C^30^. Therefore, in Mount Taishan, the growth of the forest vegetation is less affected by the temperature during the growing season. Winter low temperatures mainly affect the growth status of trees^31^. Vegetation primarily acquires carbon through leaves. Kikuzawa suggested that vegetation can maximize carbon acquisition by adjusting leaf longevity^32^. And evergreen tree species increase photosynthetic carbon harvest by prolonging leaf life span^33^. However, the combination of winter low temperatures and strong light intensifies the production of reactive oxygen species in plant leaf chloroplasts, leading to damage to the cell membrane system and, in severe cases, cell death^34^.

### Discussion on the impact of human activities on the vertical differentiation of vegetation in Mount Taishan

The forests in Mount Taishan were artificially planted following the guiding principle of “greening first, followed by improvement”, that is, afforestation is first carried out, and then the structure of forest land is optimized. Due to technological constraints, some forest stands were not planted according to the principle of vegetation adaptability to the environment, resulting in certain irrationalities in stand structure. For example, a large number of pines are selected during afforestation, resulting in a large proportion of pure forests in Mount Taishan, a decrease in the ability of forests to resist risks, and a decrease in stability. Currently, most of Mount Taishan’s coniferous forests, especially pine forests, have reached a mature or even over-mature stage. Due to the excessively high stand density, natural regeneration of pine forests is not ideal, leading to predominantly single-layer pine forests. Additionally, the high canopy closure of pine forests limits the growth of pine trees, resulting in a prevalence of “small old trees”. Consequently, many pine trees exhibit weakened vigor or even die in response to interspecies competition, environmental changes, and pest infestations^35^. To address these issues, Mount Taishan Forest Farm conducts nurturing thinning based on the principles of “promoting broadleaf trees among conifers” and “maintaining conifers among broadleaf trees”. This practice optimizes the forest structure, sustains a state of coniferous-broadleaf mixed forests, enhances the complexity of forest community structure, and improves its ability to resist adverse environmental conditions. While these measures maintain the health of Mount Taishan’s forests, they also slow down the process of broadleaf forests invading coniferous forests. Some forest technicians believe that without human-induced thinning, Mount Taishan’s forests would gradually transform into broadleaf forests^15^. And they will also lead to more mixed vegetation patches of deciduous broad-leaved forest belt and temperate coniferous forest belt, and the fuzziness and randomness of vegetation distribution in transition zone become larger.

### Advantages, limitations, and prospects of vertical belts research based on cloud model

In recent years, scholars have visually represented the topographic differentiation patterns of Mount Taishan’s vegetation by establishing Geo-informatic graphics illustrating the spatial distribution of *Pinus tabulaeformis* and *Robinia pseudoacacia*^36,37^. However, these graphics provided a general patterns of vegetation distribution, lacking the ability to portray the fuzziness and randomness within transitional zones among various vegetation types. In contrast, the cloud model approach can not only reflect the central tendencies and range of distribution heights in the core zones of vertical belts but also quantitatively describe the fuzziness of their boundaries. Nevertheless, the limitation of the cloud model lies in its emphasis on numerical patterns to describes the uncertainty in the distribution of vertical belts, resulting in a weaker connection with spatial distribution. Advancements in sensor resolution and the widespread application of technologies such as unmanned aerial vehicles (UAVs) have enhanced the precision of information conveyed by image pixels and enriched sample data accuracy^38^. This provides a data foundation for the application of cloud models. The key focus of further research is to deepen the integration between cloud models and spatial analysis methods, facilitating the development of high-precision, multi-scale vertical belt structure models. This advancement aims to offer a more intuitive representation of the distribution patterns observed within vertical belts.

## Conclusion

1. Although the distribution height of the core zone of the coniferous and broad-leaved mixed forest (EX: 1384.09 m) is higher than that of the deciduous broad-leaved forest (716.65 m) and the temperate coniferous forest (1053.51 m), the variation range of its distribution height (EN: 53.74 m) is much smaller than that of the deciduous broad-leaved forest (EN: 99.63 m) and the temperate coniferous forest (121.70 m), there is only a small range of centralized distribution.
2. The fuzziness and randomness of the distribution of the lower extension zone of deciduous broad-leaved forest and temperate coniferous forest (EN: 75.15 m and 184.56 m, HE: 24.09 m and 63.54 m) are greater than that of the upper extension zone (EN: 44.75 m and 42.49 m, HE: 14.48 m and 13.23 m). The distribution height of the lower extension zone of the coniferous and broad-leaved mixed forest varies greatly (EN: 236.74 m, HE: 47.26 m), and the distribution is more random.
3. The temperate coniferous forest exhibits greater fuzziness and randomness in distribution compared to the deciduous broad-leaved forest. In the core zones, the distribution characteristics of temperate coniferous forest and deciduous broad-leaved forest in different aspects are similar, which retains the attributes of plantation to a certain extent. But in the upper and lower extension zones, the vegetation distribution characteristics of different aspects are quite different, reflecting the adaptation relationship between vegetation and environment. At altitudes < 550 m and 900–1100 m, the EX of the distribution height of coniferous and broad-leaved mixed forest and deciduous broad-leaved forest is quite different (the difference is 22.82–39.15 m), and the difference with temperate coniferous forest is small (the difference is 4.79–7.94 m), indicating that in the upper and lower extension zones of deciduous broad-leaved forest, deciduous broad-leaved tree species have a tendency to expand to coniferous forests.

## Data Availability

The data that support the findings of this study are not openly available due to reasons of sensitivity and are available from the corresponding author upon reasonable request.
